# Clinical outcomes of boron neutron capture therapy for unresectable oral cancer: a retrospective analysis

**DOI:** 10.3389/fonc.2026.1735487

**Published:** 2026-02-13

**Authors:** Yuki Yoshino, Satoshi Takeno, Teruhito Aihara, Naonori Hu, Akinori Sasaki, Kazuhiko Akita, Yasukazu Kanai, Mai Nojiri, Tsuyoshi Jinnin, Tetsuya Terada, Shinichi Haginomori, Keiji Nihei, Koji Ono

**Affiliations:** 1 Kansai BNCT Medical Center, Osaka Medical and Pharmaceutical University, Osaka, Japan; 2 Department of Radiation Oncology, Osaka Medical and Pharmaceutical University, Osaka, Japan; 3 Department of Radiology, Kyoto Prefectural University of Medicine, Kyoto, Japan; 4 Department of Otorhinolaryngology – Head and Neck Surgery, Osaka Medical and Pharmaceutical University, Osaka, Japan; 5 Institute for Integrated Radiation and Nuclear Science, Kyoto University, Osaka, Japan; 6 BNCT Joint Clinical Institute, Osaka Medical and Pharmaceutical University, Osaka, Japan

**Keywords:** advanced stage, boron neutron capture therapy (BNCT), efficacy and safety, oral cancer, recurrent, reirradiation, unresectable

## Abstract

**Background:**

Surgery is the standard treatment for oral cancer but often causes functional and cosmetic problems, and reoperation is difficult. Radiotherapy (RT) is less effective, with reirradiation limited by normal tissue tolerance and salvage surgery after RT carrying high complication risks. Systemic therapy is used for local recurrence but yields poor outcomes, underscoring the need for better options. Boron neutron capture therapy (BNCT) is an established method that selectively delivers high tumor doses. This study evaluated BNCT efficacy and safety in unresectable oral cancers not amenable to definitive RT.

**Methods:**

This retrospective study included oral cancer patients treated with BNCT between June 2020 and June 2024 under the Japanese public health insurance system. Primary endpoints were best treatment response and incidence of adverse events (AEs), particularly severe oral mucositis (Grade ≥ 3 by Common Terminology Criteria for AEs version 5). Predictors of severe oral mucositis were also examined. Secondary endpoints included overall survival (OS), locoregional control (LRC), and progression free survival (PFS).

**Results:**

Among 74 patients (follow-up period ≥3 months), the majority (73%) had recurrent cancer. The complete response rate was 50%. The major severe acute AE was severe oral mucositis (all Grade 3) in 26% of patients. The maximum oral mucosal dose and the number of dental metals were significant predictors of severe oral mucositis. The 2-year OS, LRC, and PFS rates were 49%, 52%, and 29%, respectively.

**Conclusion:**

This study suggests that BNCT is an effective and safe treatment for unresectable oral cancers that cannot be definitively irradiated.

## Introduction

For oral cancer, surgery is the preferred treatment (1). However, surgery for oral cancer can sometimes cause functional and cosmetic issues (2), and the complex shape of the oral cavity makes reoperation difficult in recurrent cases. Radiotherapy (RT) is another treatment option, although it has been reported to be less effective than surgery (3), especially for advanced oral cancer (4). In addition, reirradiation with conventional RT is difficult because the total required dose exceeds the tolerable dose for normal organs (5–7). Salvage surgery also carries a high risk of complications after previous RT (8, 9). Therefore, systemic therapy is traditionally chosen even for locally and regionally recurrent head and neck cancers; however, it has a significantly poorer prognosis than reirradiation (10).

Boron neutron capture therapy (BNCT) can selectively deliver a radical dose to tumor cells while limiting exposure to neighboring normal tissues because the high linear energy transfer particles from a boron neutron capture reaction have a shorter range than the diameter of a single cell (11, 12). The key to this advantage is the boron drug. In clinical practice, 4-borono-L-phenylalanine (BPA) is widely used because it selectively accumulates in tumor tissue (13–16). Therefore, BNCT is a useful treatment option for unresectable cancers that cannot be definitively irradiated.

The Japanese public health insurance system approved coverage for BNCT in June 2020 based on the results of the JHN002 Phase II study, which used an accelerator-based neutron generator and Borofalan (^10^B), a BPA-based drug (17). According to this coverage, the target disease must be unresectable, locally advanced, or locally recurrent head and neck cancer. Conventional RT is preferred if it is expected to be effective.

In previous analyses of head and neck cancer after clinical BNCT, the percentage of oral cancers was 5–49% (**Supplementary Table 1**) (13, 17–20). To date, no analyses of BNCT for oral cancer have been reported. In addition, concerns have arisen regarding oral mucositis when treating oral cancer using BNCT. However, the predictors and frequency of oral mucositis remain unclear. Therefore, we analyzed the treatment response, adverse events (AEs), and prognosis after BNCT for oral cancer and identified the predictors of severe oral mucositis. To the best of our knowledge, this is the first comprehensive study on the safety and efficacy of clinical BNCT for oral cancer.

## Materials and methods

### Study design and patients

This study was a retrospective analysis of patients with oral cancer who underwent initial BNCT through the Japanese public health insurance system between June 2020 and June 2024 at the Kansai BNCT Medical Center of Osaka Medical and Pharmaceutical University (Osaka, Japan), and was approved by the ethics committee at our university (Study No. 2021-172). The study conformed to the principles of the Declaration of Helsinki (2024). Written informed consent was obtained from all patients. All patients with local primary oral cancer lesions were included in this study.

According to the rules of the Japanese public health insurance system, this study included patients for whom surgery or irradiation with conventional RT was not possible owing to a previous history. The exclusion criteria included: age < 20 years, Eastern Cooperative Oncology Group performance status (ECOG-PS) ≥ 3 at presentation, disease infiltrating the brain parenchyma, distant metastases, disease invading the carotid artery or encasing the carotid artery circumferentially, disease invading the skin or mucosa adjacent to the carotid artery, renal failure, heart failure, pregnancy, and presence of a pacemaker. Additionally, we limited eligible patients to those expected to receive curative dose treatment by pre-planning using diagnostic computed tomography (CT). In this study, the curative therapy was defined as 20 Gray X-ray equivalent dose (Gy-Eq) delivered to at least 80% of the tumor volume, based on a previous study (21). Furthermore, patients with a high risk of laryngeal edema based on the irradiated field or laryngeal findings underwent tracheotomy prior to BNCT.

The primary endpoints were the best treatment response and the incidence of AEs. Acute AEs were defined as AEs that occurred within 90 days of BNCT. Late AEs were defined as those that occurred 90 days after BNCT or were prolonged beyond 3 months. Comparative analyses were also performed to explore the risk predictors affecting severe mucositis, which was defined as Grade 3 or higher by Common Terminology Criteria for AEs (CTCAE) version 5. The secondary endpoints were locoregional control (LRC), progression free survival (PFS), and overall survival (OS). The data cutoff date was May 12, 2025.

Between June 2020 and June 2024, BNCT was performed in 300 patients at our institution, among whom 75 had oral cancer. Of these, 74 patients, excluding one patient with a follow-up period of < 3 months due to factors other than the cause of death, were analyzed for treatment response, AEs, and predictors of severe mucositis (**Supplementary Figure 1**). The evaluated predictors of severe mucositis were age, ECOG-PS score at presentation, maximum oral mucosal dose, number of dental metals in the BNCT irradiation field, and external gamma radiation dose. In this study, the assessment of dental metals was standardized across all cases. The number of dental metals within the irradiation field was counted based on a specific rule: any metal was counted if it was located, even partially, within the field, regardless of the material type. Furthermore, no dental metals were removed or modified prior to BNCT in any of the cases. Gamma radiation is produced by neutron activation on the patients after BNCT (22). In 63 patients, the external gamma radiation dose was measured using an ionization chamber survey meter ^®^ ICS-1323 (Aloka Co., Ltd., Japan) at a distance of 10 cm from the central axis skin 60 min after BNCT. Survival analysis was also performed for 74 patients, and the median follow-up period for survival analysis (range) was 13 (2–40) months.

### BNCT planning and treatment

Contouring of the gross tumor volume (GTV) and organs at risk and the generation of the treatment plans for BNCT were performed using RayStation^®^ version 9A (Raysearch Laboratories AB, Stockholm, Sweden). Dose calculations were performed using the NeuCure Dose Engine (Sumitomo Heavy Industries, Japan) (23) and Borofalan. The blood ^10^B concentration was measured by inductively coupled plasma optical emission spectrometry (ICP-OES) using an Agilent 5110 ICP-OES (Agilent Technologies, California, USA). Borofalan was administered at a rate of 200 mg/kg/h for the first two hours before irradiation, followed by 100 mg/kg/h during irradiation to maintain the boron concentration.

All plans of BNCT were single fraction. The prescribed doses were determined based on tolerable normal tissue doses. They were set to 15, 12, 9, and 5 Gy-Eq. for the skin, pharyngeal mucosa, brain, and eyes, respectively.

The treatment plans were calculated using a tumor-to-blood (T/B) ratio of 2.5, according to our previous study (19) and compound biological effectiveness (CBE) factors of 3.8, 4.9, 2.5, and 1.35 at the tumor, mucosa, skin, and other normal tissues, respectively.

### Assessment

Oral environment assessments and care of all patients were performed before BNCT by oral dentists at our institution. TNM classification and clinical stage before BNCT were determined based on the Union for International Cancer Control (UICC) TNM classification, 8^th^ Edition.

Post-treatment follow-up was performed at our institution 7 days after BNCT and every 1–3 months after BNCT. However, if the patient lived far away, the procedure was performed at the referral institution, which provided us with the follow-up information.

Treatment response and recurrence were evaluated based on the Response Evaluation Criteria in Solid Tumors (RECIST) version 1.1, via CT, magnetic resonance imaging (MRI), and/or ^18^F-fluorodeoxyglucose-positron emission tomography (FDG-PET). Macroscopic findings or histological evaluations of the biopsy were also referenced. AEs of Grade 3 or higher were assessed using CTCAE version 5. AEs that had already developed prior to BNCT were excluded.

### Statistical analysis

The Kaplan–Meier method was used to estimate median OS, LRC, and PFS. The LRC duration was defined as the period from the date of BNCT irradiation to the date of disease progression at the irradiated site, whereas the PFS duration was defined as the period from the date of BNCT irradiation to the date of disease progression or death. The log-rank test was used to compare the two groups of LRC. Comparison items for the log-rank test were clinical stage (II III IVA vs. IVB), therapy for BNCT targeting oral cancer before BNCT (systemic therapy vs. none), minimum dose of GTV (≤ 22 Gy-Eq vs. > 22 Gy-Eq; median dose = 22 Gy-Eq), GTV volume (≤ 14.8 cc vs. > 14.8 cc; median volume = 14.8 cc), therapy after BNCT before progression (systemic therapy vs. none), and cancer type (recurrence after RT vs. others).

Predictors of severe oral mucositis were assessed between covariates using Spearman’s rank correlation coefficient (Spearman’s rho). We defined a weak correlation as <0.3 and a strong correlation as >0.6. A two-sided logistic regression model was used for univariate and multivariate analyses to assess the predictors of severe oral mucositis. Only the significant predictors from the univariate analysis were used in the multivariate analysis. Categorical variables were compared using a two-sided Fisher’s exact test to identify significant cutoff values among the predictors of severe oral mucositis. The cutoff values were the points farthest from the diagonal line y = x on the receiver operating characteristic (ROC) curve under and over the median.

Statistical significance was set at *p*-value < 0.05. All statistical analyses were performed using GraphPad PRISM 10, version 10.4.1 (GraphPad Software, Boston, MA, USA).

## Results

### Patient characteristics

The patient characteristics are summarized in **Table 1**. The median age was 70 years. 37 (50%) were male. Common ECOG-PS were 0 (54%) and 1 (38%). Common primary sites were gingiva (47%) and tongue (35%). The most common histology was squamous cell carcinoma (95%). Most patients (93%) had previous RT of the head and neck region. Of these, 54 (73%) had recurrent oral cancer after RT. The most common T stage was 4a (51%). The most common N stage was 0 (68%). In context, 93% of clinical stages were advanced stage (stage III: 16%; stage IVA: 47%; stage IVB: 30%). Furthermore, 24 (32%) patients received systemic therapy prior to BNCT. Systemic therapy was administered in seven (9.5%) patients following BNCT. Five (6.8%) of these patients received immune checkpoint inhibitors (ICI). Systemic therapy was the most common treatment for recurrence after BNCT (30%). The median number of dental metals in the BNCT irradiation field was 5. Median GTV volume was 14.8 cc. Median minimum dose of GTV was 22 Gy-Eq. The median maximum oral mucosal dose was 19.5 Gy-Eq.

**Table 1:** Patient characteristics.

Patient characteristics	N (n = 74)	%
**Median age, years(range)**	70 (40–92)	
Sex, number		
Male	37	50
Female	37	50
ECOG-PS at presentation		
0	40	54
1	28	38
2	6	8
Primary site, number		
Gingiva	35	47
Tongue	26	35
Buccal mucosa	9	12
Others	4	5.4
Histology, number		
SqCC	70	95
Others	4	5.4
Past RT, number		
Head and neck cancer as the target for RT	69	93
Oral cancer as the target for RT	54	73
**Past surgery, number**	57	77
T stage, number		
0	16	22
2	8	11
3	12	16
4a	38	51
N stage, number		
0	50	68
1	4	5.4
2a	5	6.8
2b	4	5.4
3b	11	15
Clinical stage, number		
II	5	6.8
III	12	16
IVA	35	47
IVB	22	30
**Systemic therapy prior to BNCT, number**	24	32
**Systemic therapy after BNCT before progression, number**	7	9.5
Therapy for progression cases after BNCT, number		
Systemic therapy	22	30
Surgery (including endoscopic submucosal dissection)	5	6.8
definitive RT for oligometastasis	4	5.4
Photodynamic therapy	1	1.4
Secondary BNCT	5	6.8
Best Supportive Care	20	27
**Median number of dental metals in BNCT irradiation field, number (range)**	5 (2–22)	
**Median GTV volume, cc (range)**	14.8 (1.5–235)	
**Median minimum dose of GTV, Gy-Eq (range)**	22 (8.1–39.5)	
**Median maximum dose of GTV, Gy-Eq (range)**	38.8 (21.7–65.7)	
**Median maximum oral mucosal dose, Gy-Eq (range)**	19.5 (7.5–30.2)	
**Median external gamma dose 60 min after BNCT, μSV/hr (range)**	23 (5–400)	

Bold text indicates major categories. ECOG-PS, Eastern Cooperative Oncology Group performance status; SqCC, squamous cell carcinoma; RT, radiotherapy; BNCT, boron neutron capture therapy; GTV, gross tumor volume; Gy-Eq, Gray X-ray equivalent dose.

### Efficacy

**Figure 1:**
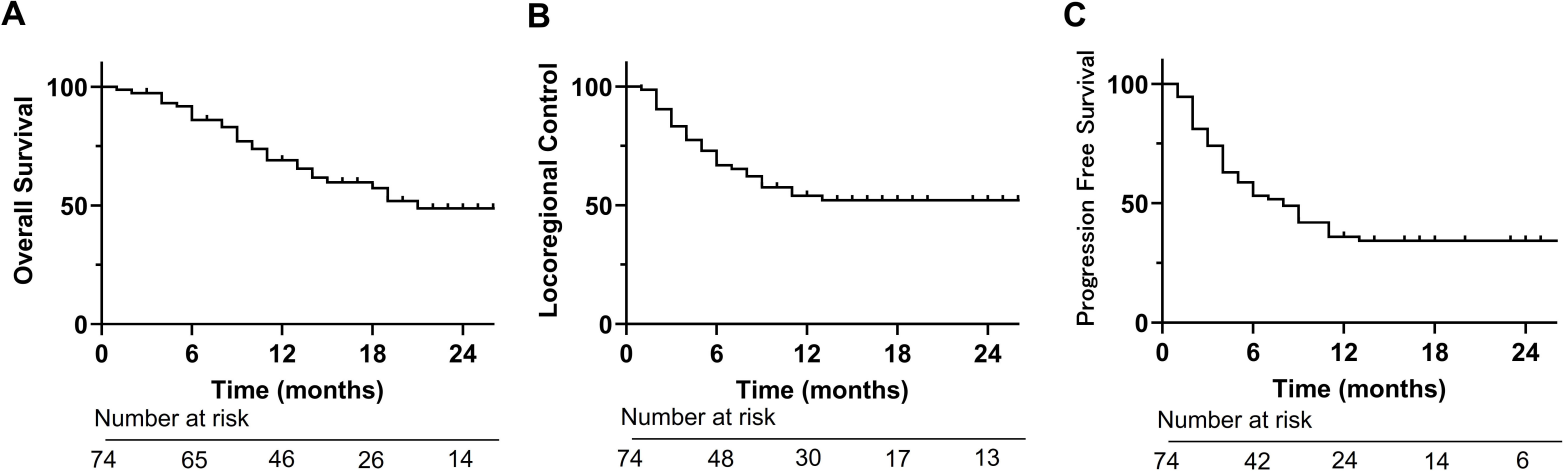
Survival. **(A)** Overall survival. **(B)** Locoregional control. **(C)** Progression free survival.

**Figure 2:**
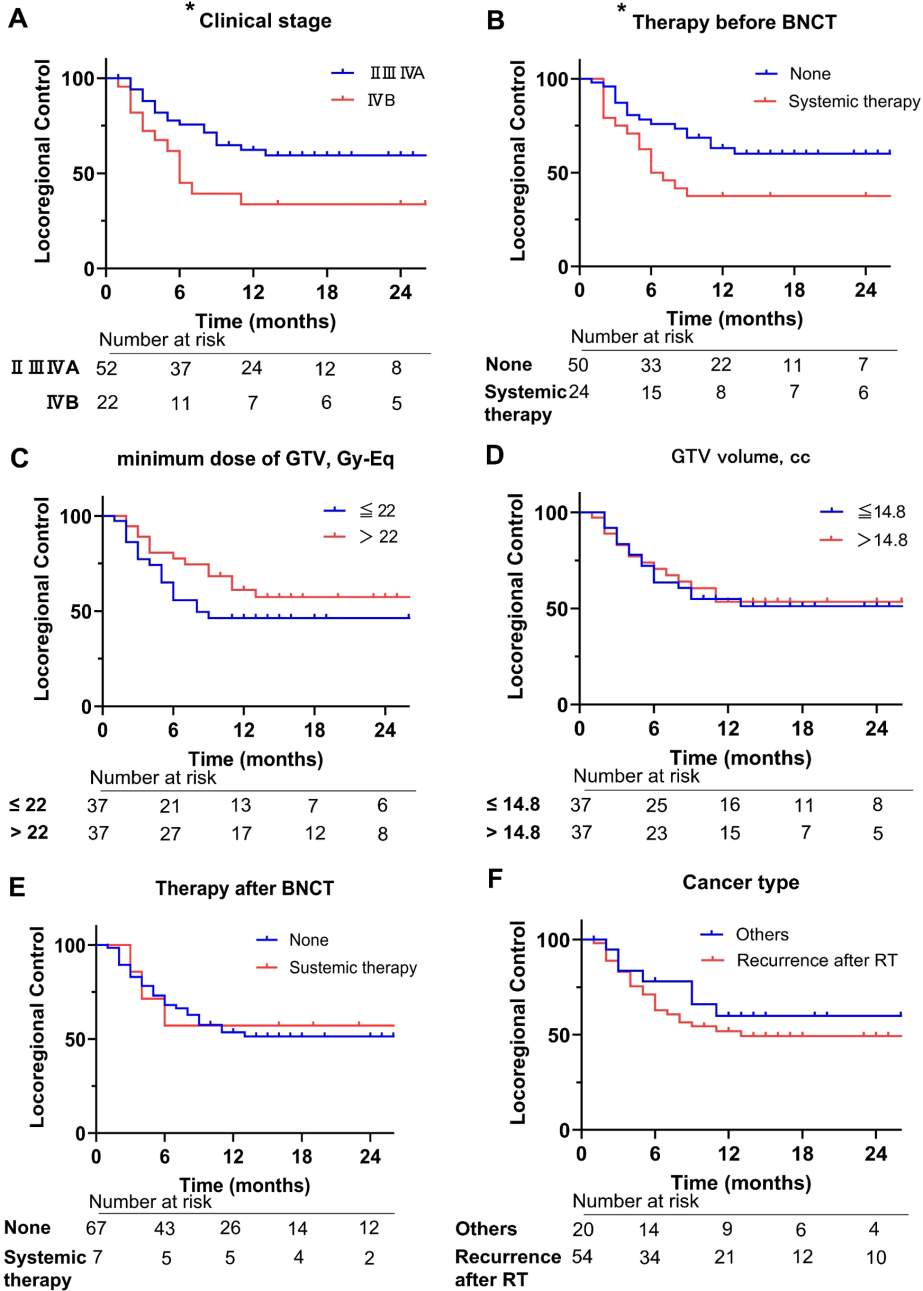
Comparison of locoregional control. **(A)** Clinical stage II, III, IVA vs. IVB. **(B)** None vs. systemic therapy prior BNCT. **(C)** Minimum dose of GTV ≤ 22 Gy-Eq vs. > 22 Gy-Eq (22 Gy-Eq = median dose of GTV). **(D)** GTV volume ≤ 14.8 cc vs. > 14.8 cc. **(E)** None vs. systemic therapy after BNCT before recurrence. **(F)** Recurrent cancer after RT vs. others (primary or recurrence after surgery). BNCT, boron neutron capture therapy; GTV, gross tumor volume; Gy-Eq, Gray X-ray equivalent dose; RT, radiotherapy. *before each title means *p*-value < 0.05.

For all 74 patients, the complete response (CR) rate was 50% (**Table 2**). The one- and 2-year OS were 69% and 49%, respectively (**Figure 1A**). The one- and 2-year LRC were 54% and 52%, respectively (**Figure 1B**). The 1- and 2-year PFS were 36% and 29%, respectively (**Figure 1C**). The log-rank test revealed that clinical stage (II/III/IVA vs. IVB) and systemic therapy before BNCT after recurrence (systemic therapy vs. none) showed significant LRC (respectively, *p* < 0.05; **Figures 2A, B**). Other comparisons did not show significant differences in LRC (**Figure 2C–F**).

**Table 2:** Treatment response.

Best treatment response	N (n = 74)	%
CR	37	50
PR	24	32
SD	6	8.1
PD	4	5.4
NE	3	2.7

CR, complete response; PR, partial response; SD, stable disease; PD, progressive disease; NE, not evaluated.

### Toxicity

Acute hyperamylasemia was observed in 34 patients (46%) as an acute Grade 3 AE (**Table 3**); however, all were asymptomatic and recovered within 3–7 days. Severe oral mucositis was observed in 19 patients (26% of all patients and 33% of patients with T1-T4 cases), all of whom were classified as having acute Grade 3 AE. Their oral mucositis were managed with hospitalization for intravenous fluid therapy and nutritional support via nasogastric tube or gastrostomy. All patients recovered and were able to resume oral intake. Kidney injury was observed in two patients (2.7%) with an acute Grade 3 AE. Skin ulceration was observed in one patient (1.4%) with an acute Grade 4 AE. Sepsis was observed in one case (1.4%) as an acute Grade 5 AE. The details of this case are provided in the Supplementary Information (see “Details and History of the Grade 5 Patient”). This patient had a history of adrenal insufficiency, and her ECOG-PS score worsened from 2 at presentation to 3 after developing pneumonia following tracheostomy for BNCT. No other patients had pre-existing adrenal insufficiency or an ECOG-PS ≥ 3 before BNCT. Soft tissue necrosis and osteonecrosis were observed in one (1.4%) and two (2.7%) cases, respectively, as a late Grade 3 AE. Hemorrhage was observed in one case (1.4%) as a late Grade 4 AE after skin ulceration as an acute Grade 4 AE.

**Table 3:** Adverse events.

Acute toxicity (n=74)	Grade 3, n (%)	Grade 4, n (%)	Grade 5, n (%)
Hyperamylasemia	34 (46)	0	0
Oral mucositis	19 (26)	0	0
Kidney injury	2 (2.7)	0	0
Skin ulceration	0	1 (1.4)	0
Sepsis	0	0	1 (1.4)
Late toxicity (n=74)	Grade 3, n (%)	Grade 4, n (%)	Grade 5, n (%)
Soft tissue necrosis	1 (1.4)	0	0
Osteonecrosis	2 (2.7)	0	0
Hemorrhage	0	1 (1.4)	0

In our study on the predictors of severe oral mucositis, we found a significant correlation between the external gamma dose and the number of dental metals (Spearman’s rho = 0.606, *p* < 0.05). However, only weak correlations were observed among the other groups (Spearman’s rho < 0.3, *p* > 0.05). Univariate and multivariate analyses revealed that the maximum oral mucosal dose and number of dental metals in the BNCT irradiation field were significant predictors of severe oral mucositis (**Table 4**). Both predictors underwent the Fisher’s exact test for severe oral mucositis (**Table 5**). Significant differences were observed in the maximum oral mucosal dose when stratified at thresholds of ≤ 16.0 Gy vs. > 16.0 Gy and ≤ 24.8 Gy vs. > 24.8 Gy (*p* < 0.05, respectively). Similarly, regarding the number of dental metals within the BNCT irradiation field, significant differences were found at cutoff values of ≤ 2 vs. > 2 and ≤ 6 vs. > 6 (*p* < 0.05, respectively).

**Table 4:** Analyses of predictors for severe oral mucositis.

Univariate analyses of predictors for severe oral mucositis used logistic regression			
	P-value	Odds ratio	95% CI
Age	0.1093	1.037	0.9941–1.087
ECOG-PS at presentation	0.3739	1.426	0.6428–3.141
* Maximum oral mucosal dose	0.0085	1.195	1.056–1.381
* Number of dental metals in the BNCT irradiation field	0.0364	1.117	1.010–1.246
Gamma dose after 60 min	0.1522	1.004	0.9985–1.009
Multivariate analyses of predictors for severe oral mucositis used logistic regression			
	P-Value	Odds Ratio	95% CI
* Maximum oral mucosal dose	0.0094	1.203	1.056–1.400
* Number of dental metals in the BNCT irradiation field	0.039	1.125	1.007–1.265

ECOG-PS, Eastern Cooperative Oncology Group performance status; BNCT, boron neutron capture therapy.

*before the predictor means *p*-value < 0.05.

**Table 5:** Analyses using Fisher’s exact test.

	Patients	Severe mucositis		P-value	Odds ratio	95% CI
	(n)	Yes	No			
* Maximum oral mucosal dose, Gy-Eq				0.0018	0	0.000–0.4403
≤ 16.0	18	0	18			
> 16.0	56	20	36			
* Maximum oral mucosal dose, Gy-Eq				0.0003	0.03504	0.003074–0.2433
≤ 24.8	66	13	53			
> 24.8	8	7	1			
* Number of dental metals in the BNCT irradiation field				0.0002	0	0.000–0.2899
≤ 2	23	0	23			
> 2	51	20	31			
* Number of dental metals in the BNCT irradiation field				0.0194	0.2558	0.08798–0.7697
≤ 6	53	10	43			
> 6	21	10	11			

Gy-Eq, Gray X-ray equivalent dose; BNCT, boron neutron capture therapy.

*before the predictor means *p*-value < 0.05.

## Discussion

Most oral cancers in this study were categorized as locally recurrent advanced oral cancers. The CR rate (50%) and the 1- and 2- year OS (69%, 49%, respectively) in this study were comparable to previous BNCT analyses of head and neck cancer (**Supplementary Table 1**) (13, 17–20). Few studies have reported the treatment outcomes of reirradiation for oral cancer (24, 25). Our 2-year LRC rate was 52%, compared to 20% in a previous statistical analysis of reirradiation for locoregionally recurrent advanced oral cancer (24). Although variations in post-BNCT treatments within our study preclude a direct comparison of OS and PFS, our 2-year OS rate was 49%, whereas other reirradiation studies for oral cancer have reported rates of 28–35% (24, 25). These findings suggest that BNCT is an effective treatment option for locally recurrent advanced oral cancers.

Comparative analyses of LRC revealed significant differences between stages II, III and IVA and stage IVB. Regarding the clinical stage, cases up to IVA are better candidates for BNCT. In several institutions, systemic therapy is the preferred treatment for recurrent oral cancer when additional curative therapies are unavailable. However, comparative analyses of the LRC also revealed significant differences between patients who received systemic therapy and those who did not. This was true not only for oral cancer in our study but also for a recent analysis of head and neck cancer overall (19). BNCT may be preferable over systemic therapy for recurrent oral cancer after curative therapy.

The meaningful major severe acute AE was severe oral mucositis (all Grade 3) in 26% of all patients and 33% of patients with non-T0 cases. The incidence of severe oral mucositis was higher in our study than in a retrospective head and neck cancer study (**Table 3**, **Supplementary Table 1**). This is consistent with the fact that oral cancer targets either the oral cavity or its vicinity. Acute AEs were not clear in the previous report of reirradiation for oral cancer (24, 25), and therefore, the incidence of acute severe oral mucositis in our study was compared to previous reports of definitive initial RT without surgery. The incidence of acute severe oral mucositis in our study was lower than that of the previous reports (CTCAE Grade 3, 44-93%; Grade 4, 0–5%) (26, 27). In our study, one patient died of acute Grade 5 sepsis. Notably, the patient had a history of adrenal insufficiency, and her ECOG-PS deteriorated from 2 at presentation to 3 before BNCT. In such a case, BNCT may act as a physiological stressor, potentially leading to further deterioration of physical condition and immune function. Therefore, careful consideration is required when determining the indications for BNCT in patients with a severely impaired baseline status. The incidence of late AEs in our study (CTCAE Grade 3: 4%; Grade 4: 1%) was comparable to that in the previous report (CTCAE Grade 3: 22%; Grade 4: 7%) (26). These results suggest that BNCT may be a safe treatment option when case selection is carefully determined.

The significant risk predictors of severe oral mucositis were the maximum oral mucosal dose and the number of dental metals. Severe oral mucositis may rarely occur if the cutoff value of the oral mucosa dose is 16 Gy-Eq or lower. However, as oral cancer is the target, it is not practical to set the maximum oral mucosa dose below 16 Gy-Eq because it must be high enough to meet the curative dose (D80 ≥ 20 Gy-Eq) of the cancer. In fact, the number of cases with >16 Gy-Eq was significantly higher when the target was a primary site (oral cavity) than when it was a non-primary site (**Supplementary Table 2**). Rather, this measure may be useful for evaluating non-oral cancers as targets. However, because the maximum dose to the oral mucosa is 24.8 Gy-Eq, it would be beneficial to ensure that the maximum oral dose does not exceed this limit if the curative dose is met.

Severe oral mucositis did not occur when the number of dental metals was two or fewer. Patients with more than two dental metals developed severe oral mucositis, even with relatively low oral mucosal doses (**Supplementary Figure 2**). Therefore, the number of dental metals can be considered a more clinically useful risk predictor than the maximum oral dose. There is still no report indicating that the number of dental metals is a clear predictor of severe oral mucositis after X-ray therapy. In BNCT, the number of dental metals may be more influential in severe oral mucositis. However, the underlying mechanism remains unclear. While it has been reported in X-ray therapy that scattered radiation increases the maximum oral mucosal dose (28), the calculated maximum dose in our study did not show a significant increase correlated with the number of dental metals. However, this does not exclude localized dose-increasing effects on the oral mucosa adjacent to the metals. This is because high-Z (high atomic number) dental materials can induce scattering and backscattering of secondary photons, leading to a highly inhomogeneous dose distribution at the metal-mucosa interface that cannot be fully captured by conventional dosimetric metrics. Although there was a correlation between the number of dental metals and the external gamma dose owing to neutron activation, the external gamma dose itself was not a significant predictor of severe oral mucositis. However, metal neutron activation also involves the emission of beta rays; therefore, it is impossible to claim that activation has no general effect. Although all patients in this study received oral care from dentists in our institution prior to BNCT, the difference in the oral environment may have affected the degree of mucositis as a difference in the number of dental metals. Removing all dental metals may be a viable treatment option to reduce risk. However, the patient’s quality of life would likely be greatly reduced, and there is no guarantee that the dental metals could be replaced after BNCT. We will continue to study the reasons for and degree of risk from dental metals, and link them to safer BNCT.

The limitations of this study are that it was retrospective, and the observation period was too short to demonstrate long-term prognosis and late AEs. Although post-BNCT treatment was not specified, the number of post-treatment cases before recurrence was small (7/74; 9.5%), and there was no significant difference in LRC compared to the no post-treatment group. However, as patients with recurrence after BNCT have multiple therapeutic options, it would be reasonable to analyze OS and PFS separately according to the treatment regimen in the future, when larger number of patients are enrolled. It is important to acknowledge that cellular uptake of BPA is known to vary across tumor models and cell lines, and is strongly influenced by L-type amino acid transporter 1 (LAT1) expression, which may be low in certain tumor types (29–31). Therefore, while BPA is currently the most widely adopted agent, future studies are warranted to identify and select the optimal boron carrier specifically for oral cancers—one that demonstrates the highest tumor-to-blood concentration ratio for this specific pathology.

## Conclusion

BNCT may be an effective treatment option for unresectable oral cancers that cannot be definitively irradiated, even when they are recurrent or advanced. In particular, patients with clinical stage up to IVA who have not undergone prior systemic therapy may be better candidates for BNCT. This may be safe when the case selection is appropriate, especially regarding baseline performance status. Severe oral mucositis is the major cause of acute adverse events. The number of dental metals was a useful predictor.

## Data Availability

The raw data supporting the conclusions of this article will be made available by the authors, without undue reservation.
