# Supplementary material for: Clinical outcomes of boron neutron capture therapy for unresectable oral cancer: a retrospective analysis
Source: Front Oncol. 2026 Feb 13;16:1735487. doi: 10.3389/fonc.2026.1735487 (PMC12945765; doi:10.3389/fonc.2026.1735487)
Supplement: Supplementary file 1 [file DataSheet1.pdf]

Supplementary Material

Clinical Outcomes of Boron Neutron Capture Therapy for Unresectable Oral Cancer: A Retrospective Analysis

Running Title: Boron neutron capture therapy for oral cancer

Authors' names and affiliations:

Yuki Yoshino, MD, PhD,[1,2,3)*], Satoshi Takeno, MD, PhD,[1,2)], Teruhito Aihara, MD, PhD,[1,4)], Naonori Hu, PhD,[1,5)], Akinori Sasaki, PhD,[1)], Kazuhiko Akita[1)], Yasukazu Kanai, PhD,[1,6)], Mai Nojiri, PhD,[1)], Tsuyoshi Jinnin, MD, PhD,[4)], Tetsuya Terada, MD, PhD,[4)], Shinichi Haginomori, MD, PhD,[4)], Keiji Nihei, MD, PhD,[1,2)], Koji Ono, MD, PhD,[1,6)]

[1] Kansai BNCT Medical Center, Osaka Medical and Pharmaceutical University

[2] Department of Radiation Oncology, Osaka Medical and Pharmaceutical University

[3] Department of Radiology, Kyoto Prefectural University of Medicine

[4] Department of Otorhinolaryngology – Head and Neck Surgery, Osaka Medical and Pharmaceutical University

[5] Institute for Integrated Radiation and Nuclear Science, Kyoto University

[6] BNCT Joint Clinical Institute, Osaka Medical and Pharmaceutical University

***Corresponding author:**

Yuki Yoshino

Email: yuki.yoshino@ompu.ac.jp

This was a retrospective analysis of the efficacy and safety of clinical boron neutron capture therapy for unresectable oral cancers that could not be definitively irradiated.

Supplementary methods

We compared the number of cases receiving a maximum oral mucosal dose of 16 Gy-Eq between the primary and non-primary site groups. Categorical variables were compared using two-sided Fisher's exact test. Statistical significance was set at p-value < 0.05.

The Grade 5 Patient's details and history

Details:

This patient was at risk of asphyxia due to bleeding associated with tumor loss after BNCT, necessitating a preemptive tracheostomy. Subsequently, she developed pneumonia post-tracheostomy, and despite recovery from pneumonia, her performance deteriorated to ECOG-PS 3. However, because of her consent and the fact that a tracheotomy was performed for BNCT and to control the pain of the oral cancer, BNCT was performed. She had a history of adrenal insufficiency secondary to immune-related adverse events

associated with pembrolizumab treatment. Unfortunately, in addition to her deteriorating performance status, the increased dose of hydrocortisone for worsening adrenal insufficiency after BNCT further compromised her immune system and led to *Candida*-induced sepsis.

History:

Left mandibular gingival cancer

- Year X: Neoadjuvant chemotherapy → Surgery (Pathology: Squamous cell carcinoma [SqCC], pT4aN0M0).
- Year X+1: Recurrence → Reoperation (Pathology: SqCC, pT2N0M0).
- Year X+3: Recurrence → Referred to our institution for boron neutron capture therapy (BNCT).

Right Maxillary Gingival Cancer

- Year X-7: Concurrent radiotherapy (self-discontinued at 42 Gy/21 fractions) for right maxillary gingival cancer; surgery (Pathology: SqCC, pT4aN2bM0).
- Year X+2: Recurrence treated with chemotherapy (5-Fluorouracil + carboplatin + pembrolizumab → paclitaxel + cetuximab) → Complete Response (CR) maintained.

Right Cheek Mucosal Cancer

- Year X-4: Surgery for right cheek mucosal cancer (Pathology: SqCC, pT1N0M0); CR was maintained.

Adrenal Insufficiency

Year X+2: Developed after pembrolizumab treatment (immune-related adverse event [irAE]) and continued hydrocortisone therapy (15 mg/day).

Supplementary Tables

Table S1. Previous studies of clinical BNCT using BPA.

Study	Year	Histology	Oral cancer	Treatment response	OS	Severe oral mucositis
**Clinical trial**						
Kankaanranta L[13]	2012	24 (SqCC), 6 (Others)	11 (37%)	CR:45%, PR: 31%	30% (2-year)	16 (53%)
K. Hirose[17]	2021	8 (SqCC), 13 (Others)	1 (5%)	CR:24%, PR: 48%	58% (2-year)	1 (5%)
**Retrospective study**						
Koivunoro H[18]	2019	79 (SqCC)	39 (49%)	CR:36% PR:32%	Cases with minimum GTV doses > 18 Gy (W): 46% (2-year)	Not evaluated
S. Takeno[19]	2024	59 (SqCC), 10 (Others)	20 (29%)	CR:44%, PR: 36%	75% (1-year)	5 (7%)
K. Hirose[20]	2024	47 (SqCC)	7 (15%)	CR:51%, PR: 23%	86% (1-year), 67% (2-year)	0

Abbreviations: SqCC = squamous carcinoma; BPA = 4-borono-L-phenylalanine; CR = complete response; PR = partial response; OS = overall survival; (W) = biological dose

Table S2. Primary site vs. non-primary site

**Maximum oral mucosal dose, Gy-Eq**	≤ 16	> 16
Primary site (in oral cavity)	7	51
Non-primary site, for example lymph node	11	5

Supplementary Figures

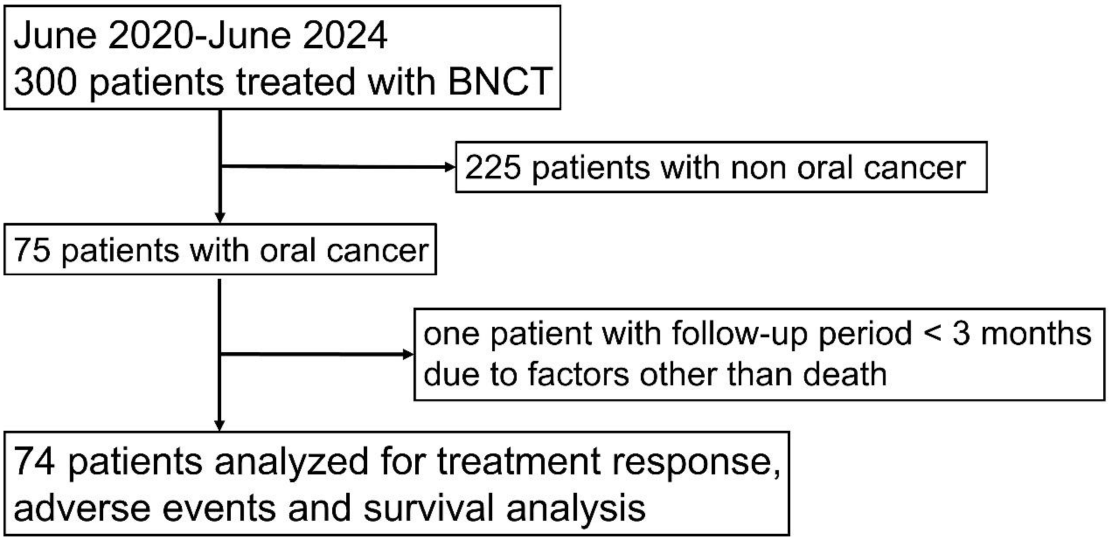

Fig. S1 Patient disposition.

The patients (n = 75) with oral cancer were treated with BNCT between June 2020 and June 2024. Of these, one patient was excluded because of a follow-up period of <3 months owing to factors other than the cause of death. Treatment response, AEs and survival were analyzed in 74 patients.

Abbreviations: BNCT, boron neutron capture therapy; AEs, adverse events.

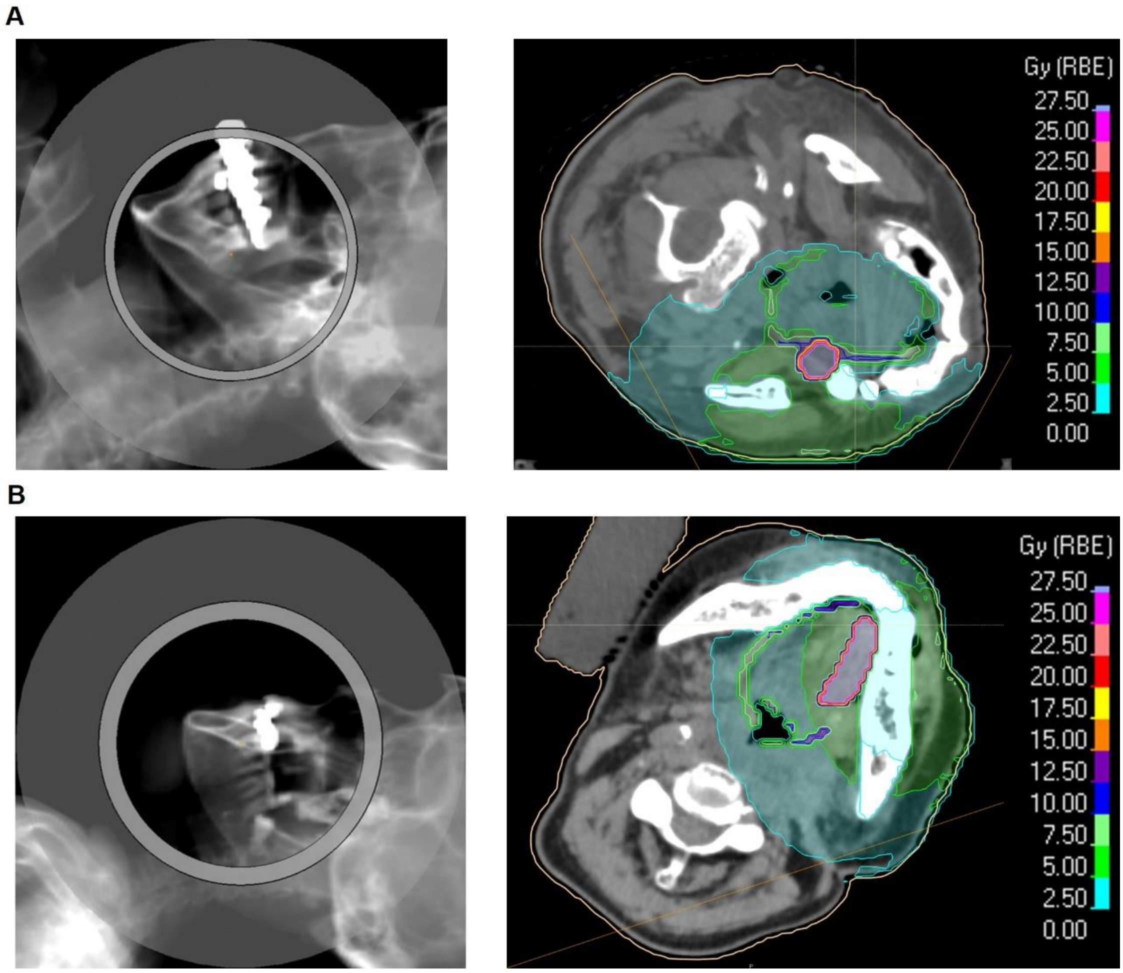

Figure S2 Representative severe mucositis cases with relatively low doses of dental metals in the oral cavity.

The left panel shows the beam eye view of the BNCT irradiation. The right side shows the isodose line of the BNCT. High dose line areas (≥ 20 Gy-Eq) correspond to almost GTV. (A) A male patient aged 84 years. The maximum oral mucosal dose is 16.1 Gy-Eq. A total of sixteen dental metals were present in the BNCT irradiation field. (B) A female patient aged 66 years. The maximum oral mucosal dose is 16.6 Gy-Eq. A total of four dental metals were present in the BNCT irradiation field.
